# Childhood Obesity: Insight into Kidney Involvement

**DOI:** 10.3390/ijms242417400

**Published:** 2023-12-12

**Authors:** Nazareno Carullo, Mariateresa Zicarelli, Ashour Michael, Teresa Faga, Yuri Battaglia, Antonio Pisani, Maria Perticone, Davide Costa, Nicola Ielapi, Giuseppe Coppolino, Davide Bolignano, Raffaele Serra, Michele Andreucci

**Affiliations:** 1 Department of Health Sciences, Magna Graecia University of Catanzaro, 88100 Catanzaro, Italy; nazareno.carullo@gmail.com (N.C.); mteresa.zicarelli@gmail.com (M.Z.); ashourmichael@yahoo.com (A.M.); teresa_faga@yahoo.it (T.F.); gcoppolino@unicz.it (G.C.); 2 Department of Medicine, University of Verona, 37129 Verona, Italy; yuri.battaglia@univr.it; 3 Department of Public Health, University Federico II of Naples, 80131 Naples, Italy; antonio.pisani@unina.it; 4 Department of Medical and Surgical Sciences, Magna Graecia University of Catanzaro, 88100 Catanzaro, Italy; mariaperticone@unicz.it (M.P.); davide.costa@unicz.it (D.C.); dbolignano@unicz.it (D.B.); 5 Interuniversity Center of Phlebolymphology (CIFL), “Magna Graecia” University, 88100 Catanzaro, Italy; nicola.ielapi@uniroma1.it; 6 Department of Public Health and Infectious Disease, “Sapienza” University of Rome, 00185 Rome, Italy

**Keywords:** glomerular filtration rate (GFR), chronic kidney disease (CKD), biomarkers, body mass index (BMI), glomerulopathy

## Abstract

This review examines the impact of childhood obesity on the kidney from an epidemiological, pathogenetic, clinical, and pathological perspective, with the aim of providing pediatricians and nephrologists with the most current data on this topic. The prevalence of childhood obesity and chronic kidney disease (CKD) is steadily increasing worldwide, reaching epidemic proportions. While the impact of obesity in children with CKD is less pronounced than in adults, recent studies suggest a similar trend in the child population. This is likely due to the significant association between obesity and the two leading causes of end-stage renal disease (ESRD): diabetes mellitus (DM) and hypertension. Obesity is a complex, systemic disease that reflects interactions between environmental and genetic factors. A key mechanism of kidney damage is related to metabolic syndrome and insulin resistance. Therefore, we can speculate about an adipose tissue–kidney axis in which neurohormonal and immunological mechanisms exacerbate complications resulting from obesity. Adipose tissue, now recognized as an endocrine organ, secretes cytokines called adipokines that may induce adaptive or maladaptive responses in renal cells, leading to kidney fibrosis. The impact of obesity on kidney transplant-related outcomes for both donors and recipients is also significant, making stringent preventive measures critical in the pre- and post-transplant phases. The challenge lies in identifying renal involvement as early as possible, as it is often completely asymptomatic and not detectable through common markers of kidney function. Ongoing research into innovative technologies, such as proteomics and metabolomics, aims to identify new biomarkers and is constantly evolving. Many aspects of pediatric disease progression in the population of children with obesity still require clarification. However, the latest scientific evidence in the field of nephrology offers glimpses into various new perspectives, such as genetic factors, comorbidities, and novel biomarkers. Investigating these aspects early could potentially improve the prognosis of these young patients through new diagnostic and therapeutic strategies. Hence, the aim of this review is to provide a comprehensive exploration of the pathogenetic mechanisms and prevalent pathological patterns of kidney damage observed in children with obesity.

## 1. Epidemiology

In recent decades, childhood obesity has reached pandemic proportions, emerging as one of the most pressing global public health concerns, similar to obesity trends in adults. The international obesity task force has categorically labeled childhood obesity as a worldwide “crisis in public health” [1]. Worldwide obesity prevalence almost tripled from 1975 to 2016, the year in which 39% of the world’s adult population were overweight and 13% obese, corresponding approximately to 1.9 billion overweight and 650 million obese [2]. According to the World Health Organization, in 2016, over 340 million children and adolescents aged 5–19 were overweight or obese with a prevalence of about 18%, and by 2020, this number had increased to 39 million children under the age of 5 [2]. Additionally, another study found a significant rise in the prevalence of age-standardized obesity. In boys, the rates surged from 0.9% in 1975 to 7.8% in 2016 and in girls from 0.7% to 5.6% over the same period [3]. However, alarming reports suggest that the prevalence of childhood overweight and obesity will continue its upward trajectory globally, with its epicenter shifting weight from Western countries to Asia and Africa, in tandem with the economic development of these areas [4]. In Africa, the number of overweight children under 5 has increased by around 24% since 2000, and in 2019, about half of the children in the same age group who are overweight or obese lived in Asia [2]. At the same time, a recent study by Inoue et al. revealed that parental household income levels can play a significant mediating role in the connection between low parental education and offspring obesity. Their analysis revealed that poverty accounted for approximately 20% of the association between low household education levels and obesity among children and adolescents in the U.S.A. [5]. Simultaneously, the prevalence of chronic kidney disease (CKD) has been on the rise, mirroring the upward trend in obesity rates [6]. The age-standardized CKD-related mortality rate increased by 41.5% from 1990 to 2017, culminating in 2017 with approximately 700 million CKD patients in all stages [7]. Notably, CKD prevalence has even escalated among children and adolescents [8,9], significantly elevating the risk of mortality within this age group [10,11]. Obesity in childhood and adolescence is strongly associated with CKD and all causes of end-stage renal disease (ESRD) [12,13]. Indeed, the prevalence of overweight and obesity in a pediatric population on renal replacement therapy in Europe was 20.8% and 12.5%, respectively [14]. Literature data on the prevalence of CKD in children are scarce. According to some records dating to 2007, the prevalence of pediatric CKD ranged from 15 to 74.7 cases per million of the age-related population [15]. CKD is frequently asymptomatic, particularly in the early stages; hence, having reliable data on CKD in the pediatric population is very difficult, and so the incidence and prevalence are likely to be underestimated [16].

## 2. Childhood Obesity Definition and BMI Related CKD Progression

In adults, the body mass index (BMI) serves as the standard measure to identify obesity as a health risk factor. However, the criteria for classifying overweight and obese individuals differ for children. According to the Centers for Disease Control, overweight children are those with a BMI above the 85th percentile, while obese children have a BMI > 95th percentile [17]. The American Heart Association defines severe obesity as having a BMI ≥ 120% of the 95th percentile or an absolute BMI ≥ 35 kg/m^2^ [18]. The baseline BMI has emerged as a critical predictor of CKD progression [19]. Research indicates that each unit increase in BMI heightens the risk of ESRD [20]. An observational study by Russo et al. underlines a significant link between high BMI and the initiation of dialysis in CKD patients. It suggested that hemodynamic changes may explain this increased risk as high BMI triggers glomerular hypertrophy and subsequent hyperfiltration, potentially expediting renal function decline and necessitating earlier dialysis initiation [21].

## 3. Impact of Obesity on Kidney Outcomes in Children

While the effects of obesity on renal function in adults have been extensively studied and well-documented, recent research is shedding light on similar trends in children. A pivotal 2017 systematic review and meta-analysis by Garofalo et al., involving over 600,000 participants from 39 cohort studies, conclusively linked obesity to an increased risk of low glomerular filtration rate (GFR) and albuminuria over a mean follow-up period of 6.8 years in adults [22]. This landmark study established BMI as an independent predictor of de novo CKD in the general adult population [22]. Further studies have reinforced these findings, emphasizing the association between abdominal obesity and the high risk of all-cause and CV mortality among ESRD patients [23]. Additionally, another large study conducted over nearly two decades (1964 to 1985) involving 330,252 individuals in the state of California in the U.S.A. demonstrated that a high BMI is a robust and modifiable risk factor for ESRD, even after adjustments for baseline blood pressure (BP) levels and the presence of diabetes mellitus (DM) [24].

In children with CKD, the evidence, while not as extensive as that in adults, suggests a similar trend. Children with obesity often become adults with obesity, thereby increasing the risk of various clinical outcomes, particularly metabolic diseases [25,26]. Obesity plays a significant role in both the onset and the progression of CKD [27,28] and is strongly correlated with the two most common causes of ESRD: DM and hypertension [29]. Metabolic syndrome, a consequence of obesity, also stands out as an independent risk factor for ESRD [30]. Notably, bariatric surgery has consistently demonstrated its ability to reduce the prevalence of metabolic syndrome [31]. In a study with a median follow-up of 13 years, children with DM had a 154% increased risk of early-onset kidney disease compared with their counterparts without DM [32]. In addition, diabetic children have an increased risk of other specific kidney diseases, including tubulo-interstitial and glomerular diseases, urolithiasis, and renal failure. The risk of developing these conditions tends to be higher in children with type 2 DM than in those with type 1-DM [32]. A consistent association has been demonstrated between increased BMI in adolescents and the development of ESRD from diabetic and nondiabetic causes. In a large study of 1.2 million adolescents with a median follow-up of about 25 years, obese adolescents showed a 3.4-fold increased risk of developing ESRD from nondiabetic nephropathy and a 19-fold increased risk of developing ESRD from diabetic causes [13].

Differently from adults, obesity-related kidney damage is believed to occur early in childhood, long before the onset of hypertension, DM, and other comorbidities commonly associated with kidney disease. Thus, obese children have been seen to have larger kidneys and increased renal flow compared with normal-weight children, supporting the idea that obesity may trigger renal changes very early in life [16].

Recent studies underscore that obesity is an independent risk factor for CKD in children [33,34], contributing to an increased risk of death among obese children with ESRD [35,36]. Moreover, even otherwise healthy overweight or obese children exhibit a markedly increased risk of ESRD later in life [13,37]. The long-term impact of overweight and obesity on CKD risk is substantial, as demonstrated by a large prospective study with 4463 participants, revealing that early-onset overweight between the ages of 26 and 36 was strongly associated with reduced renal function at age 60–64 years [38].

In one Canadian tertiary pediatric clinic, nephrology patients referred between 1985 and 2006 displayed significantly higher BMI z-scores, with obese patients having a higher risk of developing CKD later in life, surpassing predictions based solely on their primary kidney disease [39].

Surprisingly, despite the usual presence of poor nutritional status in patients undergoing renal replacement therapy, a significant proportion of the European pediatric population undergoing renal replacement therapy is overweight or obese, rather than underweight [14]. This finding carries significant health implications, as obese children with ESRD and a high BMI have an increased risk of death [40]. Therefore, nutritional management in children and adolescents in renal replacement therapy should be focused on the prevention and treatment of overweight and obesity over malnutrition. Considering these aforementioned factors, pediatric nephrologists are increasingly recognizing obesity as a serious health concern [36].

## 4. Hereditary Factors of Obesity

Obesity is a complex disease influenced by both genetic (multiple allelic variants) and environmental factors (e.g., more calorie-dense food and increasingly sedentary lifestyles) [41,42]. The interactions between genes and an increasingly obesogenic environment play a crucial role in the physiology and pathophysiology of obesity. Body adiposity has a hereditary component, but the identification of specific genes responsible for common forms of obesity has proven to be challenging. However, genomic analyses have shed light on allelic variants associated with obesity. Environmental factors, such as lifestyle changes, dietary shifts (including increased intake of sugary foods and drinks), and reduced physical activity [43], seem to modify genetic associations with BMI. Notably, severe early-onset obesity is more likely to have a predominantly genetic origin. 

In cases of monogenic forms of obesity, where genetic mutations significantly contribute to obesity, targeted therapies may be considered as part of the treatment. For example, the variants of the fat mass and obesity-associated gene (*FTO*), located on chromosome 16, exemplify the interplay between genetics and environment [44,45]. Although the exact mechanisms linking *FTO* genes to obesity remain unclear, studies indicate that they primarily affect exergy expenditure. Additionally, certain genetic variants can modify adipocyte function, influencing energy utilization and mitochondrial thermogenesis [46]. This observation has important implications for the discovery of new anti-obesity drugs. The effects of single nucleotide polymorphisms on adiposity are influenced by physical activity and macronutrient composition in the diet [47]. Other hereditary factors can independently increase the risk of conditions such as DM through various effects on different tissues [48].

**Figure 1:**
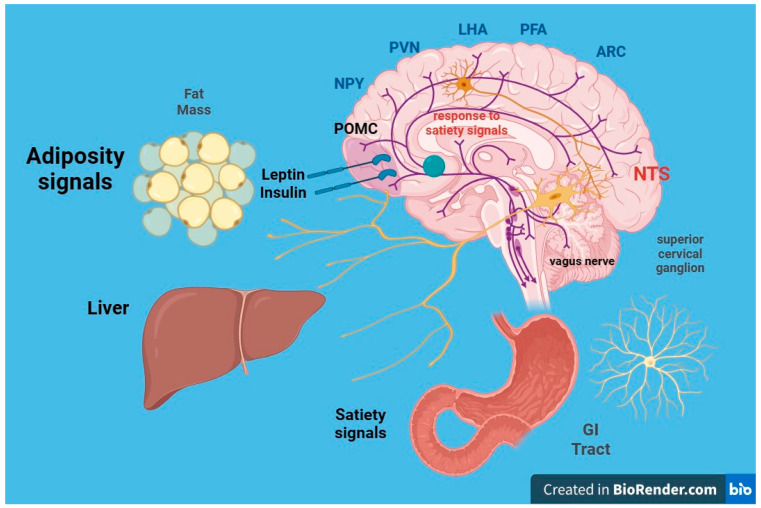
Graphical representation of some of the most important and known hereditary factors of obesity.

Several specific hereditary factors can contribute to obesity, especially in children (Figure 1):

I. leptin: Produced by adipose cells in the placenta and, to a lesser extent, in the intestine. The *Ob* (Obese) or *Lep* (Leptin) gene codes for leptin, which signals to the brain, regarding the levels of stored fat in the body. Leptin-deficient mice (*Ob* mice) show hyperphagia, insulin resistance, hyperinsulinemia, and infertility. By increasing adiposity, resistance to the action of leptin occurs. Although obesity due to leptin deficiency has been studied, most people with obesity do not have any abnormalities in the leptin gene. This suggests that obesity may be caused by either leptin deficiency or genetic defects in the leptin receptor itself [49,50].
II. Prohormone convertase 1/3 (PC1/3): a congenital deficiency of the *PCSK1* gene, responsible for proprotein convertase 1/3, can lead to a severe multihormonal disorder characterized by early-onset obesity [51].
III. Congenital deficiency of the melanocortin-4 receptor (MC_4_R): a congenital deficiency of MC_4_R is associated with early-onset obesity and above-average height [52,53,54].
IV. Proopiomelanocortin (POMC) or melanocyte-stimulating hormone (MSH): It transmits the appetite-suppressing effect of leptin through MC_4_R. Mutations in *POMC* gene can also lead to early-onset obesity due to severe hyperphagia. ACTH (AdrenoCorticoTropic Hormone) is produced by POMC in the hypothalamus, as is alpha-MSH, a key factor in reducing food intake [55,56].
V. Guanine nucleotide-binding protein G, alpha stimulant *(GNAS)* gene mutations: these are associated with Albright’s hereditary osteodystrophy (i.e., type 1 pseudohypoparathyroidism), and characterized by early-onset obesity, along with other features such as developmental delay, short stature, brachydactyly, subcutaneous ossifications, pseudohypoparathyroidism (hypocalcemia, resistance to parathormone), and resistance to thyrotropin (elevated thyroid stimulating hormone with normal or low-free thyroxine) [57].

## 5. Correlation between Low Birth Weight, Obesity, and CKD

Several early-life factors may significantly influence how obesity impacts kidney health. Researchers have explored the connection between low birth weight (LBW) and susceptibility to renal disease.

A study by Hoy et al. conducted among Aborigines in Australia’s Northern Territory, where there has been a notable epidemic of renal failure [58], revealed intriguing findings. Briefly, increasing BMI and decreasing birth weight act in concert to amplify the risk for albuminuria. In addition, LBW was associated with metabolic syndrome predisposition, as previously evidenced by the Hertfordshire study [59]. 

Preterm birth presents another critical factor in this context. It not only correlates with an increased risk of obesity development but also with reduced numbers of nephrons, which can subsequently accelerate kidney deterioration. This occurs because high body weight exerts hemodynamic and metabolic effects on each nephron, and the total number of nephrons is determined at birth [56]. The main determinants predisposing to reduced renal development appear to be caloric and protein malnutrition, placental malfunction, and maternal hyperglycemia [56]. Notably, obese children born prematurely with focal glomerular sclerosis (FSGS) face a higher risk of progression to ESRD during childhood in comparison with their obese counterparts with FSGS born at full term. It appears that preterm birth and obesity may interact, resulting in an additive risk for the progression of kidney disease in childhood [60].

Data from the CKD in Children (CKiD) study, an observational study of a large cohort of children and adolescents with mild to moderate CKD, noted a significantly higher prevalence of children who were small for gestational age and premature children compared with the general population [61]. There is evidence that prematurity and LBW are risk factors not only for the development of obesity but also for hypertension and CKD in children and adults [62].

Understanding this intricate relationship between LBW, obesity, and kidney health is essential for more targeted interventions and treatments to mitigate the risk of CKD and its progression in children and adolescents.

## 6. Neurohormonal, Metabolic and Immunological Effects of Obesity on Kidney Function

**Figure 2:**
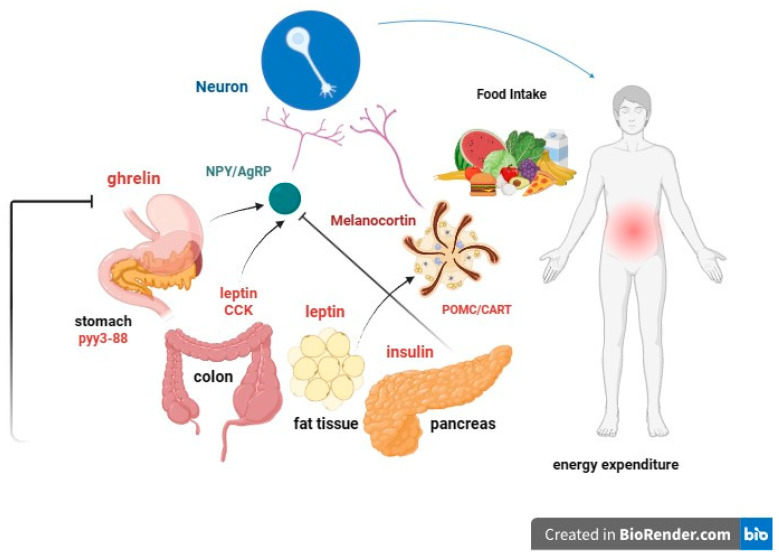
Leptin is a peptide that acts through its own receptors with intrinsic tyrosine kinase activity expressed in the arcuate nucleus of the hypothalamus. It causes the following effects: (a) Reduction in food intake and fat mass; (b) increase in energy expenditure. The leptin signal synergizes with that of insulin. Within the arcuate nucleus of the hypothalamus, two distinct populations of neurons exist: (a) Orexigenic neurons synthesize and release neuropeptide Y (NPY) and agouti-related protein (AgRP); (b) anorexigenic neurons expressing proopiomelanocortin (POMC) and cocaine and amphetamine-related transcript (CART) synthesize and release melanocortin (MCH) and other transmitters. Leptin decreases the synthesis of NPY and AgRP in anorectic neurons and increases the synthesis of MSH in POMC neurons. From the arcuate nucleus, orexigenic and anorexigenic neurons project to the paraventricular nucleus (NPV) of the hypothalamus, regulating energy expenditure, sympathetic response, and thermoregulation.

The intricate neurohormonal mechanisms responsible for obesity have been subjects of extensive research for years (Figure 2). There is ongoing debate about whether obesity and its associated metabolic syndrome directly induce kidney damage. Although causality remains to be confirmed by cause-and-effect studies, a growing body of epidemiological studies and clinical observations suggests that the metabolic syndrome associated with obesity can indeed play a key role in the development of CKD [63]. Otherwise, CKD leads to many endocrine and immunological abnormalities in adipose tissue.

However, despite the demonstrated correlation between obesity, metabolism, and renal insufficiency, it is crucial to note that not all overweight patients develop CKD. In fact, approximately 25% of obese individuals are metabolically healthy, implying that weight gain alone may not be the sole trigger for the development of kidney disease [64]. 

Obesity, nonetheless, causes metabolic alterations that can contribute significantly to the onset and progression of kidney disease. Furthermore, obesity has been shown to independently influence the CKD process, even in conditions such as patients with unilateral renal agenesis [65] or after unilateral nephrectomy [66]. 

This complex scenario of multiple bidirectional interactions between adipose tissue and the kidney defines a specific adipose tissue–kidney axis.

CKD causes a redistribution of body fat with a reduction in subcutaneous fat volume and an increase in visceral and ectopic fat deposits in skeletal muscles and the liver, with a consequent harmful effect known as lipotoxicity [67]. In CKD, lipid deposition also occurs in the kidney and results in increased renal inflammation [67]. 

Additionally, it has been observed that obesity is commonly associated with a state of subclinical, low-grade inflammation in adipose tissue. Indeed, a recent study has shown that the exposure of adipose tissue to uremic serum was able to activate NF-κB (nuclear factor kappa-light-chain enhancer of activated B cells) and HIF-1α (hypoxia-inducible factor 1-alpha), resulting in adipose tissue inflammation. As evidence of the latter, adipose tissue from dialysis patients had high levels of markers of inflammation [68], suggesting that it may be a source of the chronic low-grade subclinical inflammation observed in CKD patients in a manner unrelated to excess adiposity [69]. CKD patients exhibit markedly higher levels of leptin, IL-6/IL-10 ratios, tumor necrosis factor, and high-sensitivity C-reactive protein than healthy individuals [70]. Moreover, CKD promotes macrophage infiltration of adipose tissue, which also determines an inflammatory state and the consequent development of insulin resistance and glucose intolerance [67,71,72]. It has recently been demonstrated that ESRD patients have a significantly higher density of adipose tissue macrophages compared with healthy subjects [73]. Another alteration that can be observed in obesity is fibrosis of adipose tissue. The fibrotic milieu of the extracellular matrix also contributes to the inflammatory events that accompany adipocyte hypertrophy [74,75]. There are several matrix metalloproteinases (MMPs) that regulate extracellular matrix remodeling and inflammation in adipose tissue, and among them, MMP-12 is related to renal dysfunction [76].

Adipose tissue, now recognized as an endocrine organ, plays a pivotal role in these metabolic effects. It secretes various cytokines called adipokines, which play a critical role in the development of obesity complications [77]. These adipokines have direct implications for lipid metabolism, inflammation, immune response, insulin resistance, atherosclerosis, metabolic homeostasis, and cell migration and proliferation [78]. More than 600 proteins secreted by adipocytes have been recognized, including tumor necrosis factor, IL-6, fatty acid binding protein 4, chemerin, angiotensinogen, adiponectin, and leptin [79,80,81]. The accumulation of urea in CKD also determines an increase in oxidative stress in adipose tissue, which results in an increase in the production of some adipokines such as resistin and retinol-binding protein-4, which contribute to the onset of insulin resistance [72].

Cytokines produced by the adipose tissue in obese patients can induce both adaptive and maladaptive responses in renal cells, potentially compromising glomerular function through hyperfiltration [77]. Several synthesized adipokines, including but not limited to leptin, adiponectin, vascular endothelial growth factor, angiopoietins, and resistin, play crucial roles in extracellular matrix accumulation, ultimately culminating in renal fibrosis [82]. Other pro-inflammatory cytokines secreted by adipose tissue can activate endothelial cells and leukocytes at the level of renal microcirculation, altering endothelial barrier function and possibly leading to irreversible tubular damage with nephron loss. Notably, tumor necrosis factor-α and IL-6 have been found to be associated with CKD [83]. Adipokines also indirectly damage the kidney by contributing to the development of insulin resistance and hypertension [84].

Recognition of adipose tissue mediators that cause kidney damage has a relevant clinical implication as it could allow the identification of new targets for pharmacotherapies and the conception of models for predicting the risk of CKD/ESRD in obese subjects.

LEPTIN (adipose tissue) and INSULIN (pancreas) act as long-term signals of energy reserve, while GRELIN and other peptides act as short-term signals. Reductions in fat mass correlate with decreases in plasma leptin concentrations and insulin resistance. In obese individuals, circulating leptin levels are elevated and associated with leptin resistance. The leptin receptor is also expressed in the gonads, influencing sexual maturation and reproduction. Food deprivation suppresses the hypothalamic–pituitary–gonadal axis, leading to anorexia and prolonged fasting.

### 6.1. Insulin Resistance

Obesity is frequently accompanied by insulin resistance in peripheral tissues, resulting in hyperinsulinemia. Insulin itself exerts effects on the kidneys, either directly or indirectly, through the mediation of pro-inflammatory cytokines. 

A cross-sectional analysis of data from the National Health and Nutrition Examination Survey (1999–2004), involving 2515 adolescents aged 12 to 19, unveiled noteworthy insights. Among overweight adolescents, as opposed to those adolescents with normal weight, a significant association was observed between microalbuminuria and impaired fasting glucose, highlighting the link between insulin resistance and CKD in this population [85].

### 6.2. Leptin

Leptin, a hormone primarily metabolized in the kidneys, exerts its effect by binding to the megalin endocytic multiligand receptor in the renal proximal tubule [86]. Notably, serum levels of leptin, a proinflammatory adipokine, are approximately 5–10 times higher in obese individuals than in non-obese ones [87]. 

Leptin’s functions extend beyond the regulation of appetite, energy expenditure, and body weight. It also impairs the immune system and may exacerbate renal dysfunction [88] by inducing glomerular hypertrophy and proliferation of glomerular mesangial and endothelial cells [89,90]. That is because leptin can act on different intrarenal signaling pathways, as glomerular endothelial cells and mesangial cells abundantly express leptin receptors. Leptin promotes the expression of pro-fibrotic genes, such as TGF-β1 (transforming growth factor-β1) and pro-inflammatory cytokines. TGF-β1 can also bind to its renal receptors and increase the expression of other pro-fibrotic factors in a positive feedback loop. Furthermore, TGF-β1 is a potent stimulator of mesangial cell proliferation. Thus, the action of these molecules can result in glomerular basement membrane thickening, mesangial matrix accumulation, and mesangial hypertrophy, determining, in turn, glomerulosclerosis and proteinuria [91].

Importantly, leptin levels show strong correlations with inflammation and insulin resistance, both of which are recognized risk factors for the development of CKD [92,93].

Finally, leptin is also considered a uremic toxin that could contribute to the onset of several complications of CKD, such as cachexia, protein-energy wasting, hypertension, cardiovascular disease, and bone diseases [94].

### 6.3. Adiponectin

Adiponectin is regarded as a predictor of chronic renal failure, and its role in safeguarding against metabolic complications associated with obesity is notable. This anti-inflammatory adipokine plays a protective role in mitigating insulin resistance and lipid accumulation [95]. It also has inflammatory and anti-apoptotic properties, probably through the activation of AMP-activated protein kinase signaling [91,96]. Interestingly, it was observed that adiponectin knockout mice develop microalbuminuria, kidney fibrosis, oxidative stress, inflammation, and podocyte dysfunction, whereas restoration of adiponectin levels rescues renal function [97].

Surprisingly, levels of adiponectin are increased in patients with renal impairment. Indeed, circulating adiponectin levels exhibit an inverse relationship with both the percentage of body fat [98,99] and the estimated GFR value [100]. Furthermore, low plasma adiponectin levels are inversely correlated with proteinuria in obese subjects and may predict adverse renal outcomes in patients with type 2 DM [101,102]. 

### 6.4. Other Adipokines

Visfatin and resistin display pro-inflammatory and atherogenic effects. Visfatin promotes the expression of TGF-β1, plasminogen activator inhibitor-1, and type I collagen, having an important pro-fibrotic role, whereas resistin stimulates the production of ICAM-1 (intercellular adhesion molecule-1) and VCAM-I (vascular cell adhesion molecule-1) and promotes the activation of the sympathetic renal system. Levels of these adipokines are increased in obesity and CKD and are associated with reduced GFR [91]. Adipose tissue also produces components of the renin–angiotensin–aldosterone system (RAAS). Adipocyte-specific deletion of angiotensinogen in mice reduces plasma levels sufficiently to decrease systolic BP. Moreover, adipocytes synthesize and secrete aldosterone, which is increased in obese animals [103]. Increased RAAS activation, in conjunction with glomerulomegaly and impaired sodium/glucose reabsorption, results in hypertension and glomerular hyperfiltration [104].

## 7. Hypertension and Obesity in Children

Hypertension associated with obesity is a complex multifactorial disease, including activation of the RAAS, impaired vascular function, and increased sympathetic nervous system (SNS) activity [105]. While a well-established relationship exists between obesity and hypertension in adults, the prevalence of hypertension and pre-hypertension in the pediatric setting is significantly higher among obese children and adolescents [106,107]. The kidney is both a cause and victim of hypertension [108] because, on the one hand, the presence of reduced insulin sensitivity, dysglycemia, and dyslipidemia (both precursors of hypertension) can lead to renal damage, and on the other hand, functional and structural alterations in the nephrons can contribute to increased BP [109].

Hypertension is not only a risk factor for the progression of renal disease in the pediatric population with CKD [110], but it is also a significant contributor to renal dysfunction in obese patients, although it is not the sole hemodynamic cause [30]. Studies have shed light on the profound impact of adiposity on BP levels in the pediatric population. For instance, research by Tu et al. [111] revealed a four-fold increase in BP levels across all age groups in small patients with a BMI > 85th percentile. Similarly, Sorof et al. [112] demonstrated a progressive increase in hypertension prevalence in school-aged children as BMI exceeded the 95th percentile. 

Furthermore, an Italian study showed that prehypertensive children exhibited reduced GFR and increased proteinuria compared with normotensive children [113]. High-normal BP levels in the pre-hypertension group correlated with low-normal renal function, characterized by decreased GFR and increased proteinuria levels, albeit still within accepted normal ranges. Notably, left ventricular hypertrophy is more common in children with stage 2–4 CKD and with masked or confirmed hypertension [114], underlying the importance of identifying hypertension in these cases.

Masked hypertension has emerged as a particularly strong independent predictor of left ventricular hypertrophy in the pediatric population, likely attributed to the hyperactivation of the RAAS [115]. The correlation between arterial hypertension and obesity in children involves several factors. These include insulin resistance, either alone or combined with hyperleptinemia, which activates the SNS, leading to vasoconstriction, reduced renal blood flow, and subsequent activation of RAAS and water and sodium retention [116]. 

Elevated leptin levels also contribute to increased BP and SNS activation. In the pediatric population, proinflammatory cytokines, oxidative stress pathways, sleep apnea syndrome, or poor sleep quality can further exacerbate arterial stiffness and endothelial dysfunction [117], increasing the risk of hypertension in obese children [118]. Moreover, low serum levels of 1,25-dihydroxyvitamin D (1,25-(OH)_2_D_3_) have been associated with metabolic syndrome and hypertension in Caucasian children and adolescents [119].

Hyperuricemia, which can result from a high fructose diet and increased uric acid production by adipose tissue in obese individuals, also represents a potential correlation mechanism between hypertension and obesity [120]. Studies in Moscow have shown hyperuricemia (>8.0 mg/dL) in a higher percentage of children with hypertension compared with those with normal BP [121]. Large-scale epidemiological studies are needed to further confirm these findings.

## 8. Renal Biomarkers in Obese Children

CKD and renal injury due to obesity are frequently asymptomatic in the early stages and difficult to recognize, especially in the pediatric population, where individuals often remain underdiagnosed for several years. Therefore, reliable biomarkers are needed to prevent potentially very serious renal complications and dramatically improve the overall management of this disease. Traditional renal biomarkers include serum creatinine (sCr), blood urea nitrogen (BUN), urinary albumin/protein, and volume excretion values. However, sCr and BUN have limitations in distinguishing renal injury from hemodynamic changes, especially in acute kidney injury. Furthermore, their levels may not rapidly change in response to damage due to the presence of a functional reserve mechanism in nephrons. This “reservoir” consists of other nephrons that can increase their own function in response to injury. As a result, sCr and BUN levels often remain within the “normal range” until substantial injury has occurred, potentially leading to irreversible loss of a significant number of nephrons. Among the many equations available for estimating GFR, it has been seen that FAS age (height-independent full-age spectrum equation), FAS height (height-dependent full-age spectrum equation), and LMR18 (adjusted-creatinine revised Lund-Malmö equation) are the preferred serum creatinine-based formulas in children with overweight or obesity [122]. While measurements of GFR using methods such as sCr or iohexol clearance provide important insights into renal function, they may not closely parallel the extent of the renal lesion. 

In recent years, several studies have been conducted with the aim of identifying new biomarkers for early detection of kidney injury, although most of these are used exclusively in research settings and need further validation. Some of these (Table 1) include urinary glutamyl aminopeptidase (GluAp), urinary podocalyxin (PCX), urinary kidney injury molecule-1 (KIM-1), alpha-1-acid glycoprotein (AGP), urinary N-acetyl-beta-D-glucosaminidase (NAG), and urinary neutrophil gelatinase-associated lipocalin (NGAL) [123,124]. Overexpression of these biomarkers was evidenced in the obese pediatric population [125,126]. Thus, at present, there are very few human studies concerning biomarkers of early kidney damage secondary to obesity, particularly in pediatric populations. 

In obese children and adolescents, diagnostic screening strategies could be considered in order to identify any early functional and/or structural kidney injury and consequently reduce the likelihood of potential complications, improving their prognosis. However, currently, there is insufficient evidence to recommend screening for kidney complications in non-diabetic and non-hypertensive children and adolescents with obesity [127].

Cystatin C is another marker used for estimating GFR and is independent of muscle mass in children [128,129]. Although some studies, such as those by Miliku et al., suggest that both eGFR derived from creatinine (eGFR_creat_) and cystatin C (eGFR_cystC_) blood concentrations can be influenced by factors such as BMI and body surface area, it is worth noting that eGFR_creat_ is more strongly affected by lean mass percentage and fat mass percentage [130]. Urinary cystatin has been found to be useful in detecting early kidney injury due to obesity in children [124].

An alternative biomarker for estimating renal function is rescaled serum creatinine (SCr/Q), which accounts for sex, age, or height. SCr/Q and almost all GFR estimations correlated with obesity-related comorbidities (anthropometric and metabolic variables) in 600 children with overweight and obesity without overt kidney disease [131]. Nevertheless, the progression of renal dysfunction in obesity often starts with microalbuminuria, which can eventually progress to overt proteinuria. A Framingham study has shown that overweight and obese individuals were more likely to develop proteinuria when compared to healthy individuals [132]. One dramatic demonstration of the impact of obesity on renal function was observed in patients who underwent unilateral nephrectomy. Among obese nephrotic patients, 92% developed proteinuria and impaired renal function over a 10-year follow-up period, whereas only 12% of non-obese subjects experienced such complications [133]. 

As mentioned previously, microalbuminuria functions as both an early marker of CKD and a marker of renal damage in non-diabetic patients. Csernus et al., for instance, showed that elevated levels of albuminuria and β2-microglobulinuria in obese children, compared with children with a normal body weight, suggest early renal glomerular and tubular damage, resulting from obesity [134]. Ferris et al. have also highlighted that microalbuminuria is strongly correlated with the severity of obesity in adults [135]. The use of multiple markers in nephrology will be useful as they can provide diverse information, including insights into the site of the lesion, the presence of inflammation, and any association with systemic diseases.

Alpha-1-acid glycoprotein (AGP) is an acute-phase protein that has both pro- and anti-inflammatory actions. Medyńska et al. [136] have observed that α1-AGP increased before the onset of albuminuria, suggesting that it could be a biomarker of early glomerular damage in obese children.

Lipocalin associated with neutrophilic gelatinase (NGAL) is one of the intriguing biomarkers in this context. NGAL is a 25 kDa protein associated with neutrophilic gelatinase, and it is known to be released by renal tubular cells damaged in acute kidney injury even before a decrease in GFR occurs [137]. Studies have identified NGAL concentrations in serum and urine as independent predictors of CKD progression [138] in patients with moderate renal disease, and it has been noted that its urinary concentrations may predict the development of CKD in obese adolescents with normal or reduced GFR [139]. Furthermore, NGAL is currently employed as an early biomarker for diabetic nephropathy [140]. Notably, in a study by Goknar et al., urine NGAL concentrations did not show significant differences between obese children and healthy controls [126]. However, Şen et al. observed that obese children with insulin resistance had elevated urinary levels of NGAL [141]. Thus, these investigations showed mixed results regarding the possible role of NGAL as a biomarker of early kidney damage due to obesity.

Another promising biomarker is kidney injury molecule-1 (KIM-1), a transmembrane glycoprotein whose protein expression is typically absent in a healthy kidney but can be detected in the urine of patients with AKI. KIM-1 has gained importance as a valuable biomarker for renal tubular injury [142]. For instance, in a case-control study involving 40 children undergoing cardiac surgery, urinary levels of KIM-1 were significantly increased at the 12 h timepoint [143]. The findings from the study by Goknar et al. also indicated that obese children had higher urinary KIM-1 levels compared with healthy controls [126]. Collectively, these results suggest that KIM-1 could be a potential screening biomarker for the early detection of kidney damage in obese children.

Podocalyxin (PCX) is the main surface antigen of podocytes and correlates positively with glomerulosclerosis progression and glomerular injury severity. Suwanpen et al. [144] found that PCX was increased in obese patients compared with normal-weight subjects, suggesting tubular damage. Therefore, it may be a potential biomarker of early kidney injury in obesity [144,145].

Megalin is a constitutive proximal tubule cell protein and has been shown to be a useful biomarker of obesity-related glomerulopathy (ORG) and renal injury in children [146].

Other new potential markers that detect specific early glomerular and podocyte changes are urinary podocin and nephrin [147,148,149].

**Table 1:** Potential biomarkers for detection of early kidney injury caused by obesity. Table adapted from “Obesity-related glomerulopathy in children: connecting pathophysiology to clinical care” [150].

Proximal Tubular Injury Markers	Distal Tubular Injury Markers	Glomerular Injury Markers
Cystatin C	N-acetyl-beta-D-glucosaminadase (NAG)	Cystatin C
N-acetyl-beta-D-glucosaminadase (NAG)	Neutrophil gelatinase-associated lipocalin (NGAL)	Podocin
Neutrophil gelatinase-associated lipocalin (NGAL)	Alpha-1-acid glycoprotein (AGP)	Nephrin
Alpha-1-acid glycoprotein (AGP)		Podocalyxin (PCX)
		Alpha-1-acid glycoprotein (AGP)

## 9. Proteomic and Metabolomic Approaches to the Discovery of Novel Biomarkers of Kidney Injury

Developing biomarkers that directly reflect renal lesions and are easily measurable from readily obtainable body fluid, such as blood or urine, could change the paradigm to facilitate direct monitoring of kidney health. Innovative technologies, such as proteomics and metabolomics [151,152,153,154], have identified polypeptides and metabolites as potential biomarkers for kidney disease. These biomarkers can provide early insights into kidney damage and disease progression. 

Using urinary proteome analysis, Decramer et al. [155] successfully predicted the clinical evolution of infants with ureteropelvic junction obstruction several months in advance, achieving an impressive accuracy rate of 94%. Benito et al. [152] discovered that five metabolites, including glycine, citrulline, asymmetric dimethylarginine, and symmetric dimethylarginine, were significantly increased in pediatric patients with CKD, regardless of their creatinine level, and one other metabolite, namely dimethylglycine, was also increased in patients with high creatinine levels, suggesting these as potential biomarkers of renal impairment. In another study [154], five metabolites were also found to be increased in plasma from pediatric patients with CKD, whereas bilirubin was significantly decreased. Sharma et al. used metabolomic approaches to discover that at least 13 metabolites were significantly involved in their patient cohorts, with 12 of these metabolites being related to mitochondrial metabolism. This finding suggests a global suppression of mitochondrial activity in diabetic kidney disease [156]. Sood et al. [157] conducted a population-level cohort study using metabolic profiles from 1,288,905 newborns from 2006 to 2015 to detect the metabolic profiles at birth potentially associated with a higher risk of CKD or dialysis. For CKD, the strongest associations were with citrulline, phenylalanine/glycine, acylcarnitine, acylcarnitine ratios, and the ratios between amino acids and acylcarnitine to endocrine markers such as 17-hydroxyprogesterone. For dialysis, the strongest associations were with amino acid ratios (phenylalanine/glycine, phenylalanine/tyrosine, citrulline/tyrosine), acylcarnitine ratios, and the ratio of amino acids to acylcarnitine [157]. With the same purpose in mind, other investigators, in a study on 645 children with CKD, recognized seven plasmatic metabolites that were significantly associated with CKD progression: N6-carbamoylthreonyladenosine, 5,6-dihydrouridine, pseudouridine, C-glycosyl tryptophan, lanthionine, 2-methylcitrate/homocitrate, and gluconate. Among those with eGFR < 60 mL/min/1.73 m^2^, higher levels of tetrahydrocortisol sulfate were associated with a lower risk of CKD progression [158,159].

Wendt et al. [160] investigated the association between urinary peptides, BMI, and renal function in proteome data sets from 4015 individuals. A total of 365 urinary peptides were identified to be significantly associated with BMI. Most of these peptides were collagen fragments, and most of them also demonstrated a significant concordant association with eGFR. These data strongly suggest that, on a molecular level, there may be a relationship between obesity and fibrosis, which may be among the factors responsible for kidney damage secondary to obesity.

Other significant examples of biomarkers for kidney damage include proteins, lipids, microRNAs, genomic biomarkers, imaging biomarker assessments, electrical signals, and cells present in urinalysis.

## 10. Obesity: Mechanisms of Kidney Damage

The exact mechanisms underlying obesity-related kidney damage remain unclear and are believed to initiate with a phase of hyperfiltration [161], serving initially as a physiological adaptation of the kidney to increased body mass [162]. This chronic state of hyperfiltration subsequently amplifies progressive renal damage, characterized by increased protein loss and culminating in a phase of glomerulomegaly, cell remodeling, and fibrotic scarring [163]. Presumably, a variable combination (Table 2) of hemodynamic and metabolic changes and lipid nephrotoxicity may cause or aggravate kidney injury in obese patients [164].

Obesity is widely recognized as a low-grade inflammatory state: Adipose tissue, particularly visceral fat, is a major source of endocrine bioactive pro-inflammatory compounds, while systemic levels of anti-inflammatory adipokines are reduced. Emerging evidence suggests that this pro-inflammatory state, along with increased levels of oxidative stress and an overactive RAAS, could play a role among the underlying mechanisms linking obesity with altered metabolic states, vascular dysfunction, cardiovascular disease, and even kidney changes. These potential connections between obesity and kidney disease warrant further investigation, especially in children, in order to facilitate the development of preventive strategies aimed at halting the progression of CKD [36].

One of the primary mechanisms of kidney damage in obese patients is lipid toxicity. The prevalence of dyslipidemia in CKD cohorts is significantly higher than that of the general population. Notably, obese children have higher lipid and lipoprotein levels compared with their normal-weight counterparts. For every 10 mL/min/1.73 m^2^ of GFR decline, there is a positive correlation with triglycerides and non-HDL cholesterol levels and a negative correlation with HDL cholesterol levels. 

Dyslipidemia is also independently associated with nephrotic-range proteinuria. Dyslipidemia is well established as a cardiovascular risk factor in CKD adults, and statin therapy has been demonstrated to improve lipid levels, decrease proteinuria, and potentially exert a protective effect on renal disease progression in CKD adults [178]. Pediatric kidney transplant patients preventively treated with pravastatin [179] in the immediate post-transplant period exhibited a significant decrease in total cholesterol, triglycerides, LDL levels, and even HDL levels compared with controls. While emerging data point to the efficacy of hyperlipidemia treatment in pediatric patients, there is still a lack of data concerning the influence of statin treatment on vascular disease development, progression of renal failure, or improved survival rates in children with chronic renal insufficiency. As described above, recent evidence also supports the hypothesis that reduced insulin sensitivity and hyperinsulinemia are among the most important factors contributing to kidney damage [180].

Lipotoxicity contributes to atherosclerosis and organ dysfunction, including kidney disease, through the involvement of intracellular accumulation of non-esterified free fatty acids (FFA) and triglycerides [181]. Abdominal adipose tissue generates high levels of circulating FFA. Factors such as hypoadiponectinemia, leptin resistance, and cytokine release from accumulated adipose tissue and macrophages hinder the uptake of FFA by mitochondria in various tissues, reducing FFA oxidation and promoting its intracellular accumulation [30]. Nephrotoxicity attributed to lipid accumulation in renal tissue may arise as a result of various mechanisms, including structural and functional changes in glomerular and tubular epithelial cells, leading to the development of obesity-related glomerulopathy [96,182]. Subsequently, mesangial and epithelial renal damage promote the progression of renal disease [183]. Furthermore, in podocytes, this may interfere with the insulin pathway that is crucial for the survival and maintenance of podocyte structure, causing podocyte apoptosis [184] and inducing a compensatory hypertrophic response in the remaining podocytes [104]. The presence of proteinuria, which results in tubulointerstitial and glomerular damage, is likely the consequence of fatty acid-induced lipotoxicity when albumin-saturated FFAs are excessively filtered and reabsorbed by endocytosis [182].

Furthermore, increased production of reactive oxygen species and lipid peroxidation, mitochondrial dysfunction, and tissue inflammation represent pivotal mechanisms underlying damage to podocytes, proximal tubular epithelial cells, and tubulo-interstitial tissue, ultimately resulting in glomerular and tubular lesions [171]. It is important to note that renal proximal tubular cells are particularly vulnerable to lipid toxicity due to their increased energy expenditure.

## 11. Cancer, Stones, and Gout: Impact of Obesity on Kidneys

A meta-analysis of 17 observational studies revealed a concerning trend: A higher BMI was associated with an increased risk of renal cell cancer in both men and women [111]. Additionally, the prevalence of nephrolithiasis is on the rise in parallel with the increasing prevalence of obesity. In the U.S.A., the prevalence of stone disease surged from 3.8% in the 1970s to 5.2% in the 1990s [23]. Metabolic syndrome, commonly linked with obesity, triggers alterations in urinary composition, resulting in elevated levels of uric acid, oxalate, and calcium, along with decreased levels of citrate. Consequently, there is a 75% increase in the formation of uric acid and calcium oxalate stones [24].

‘‘Gouty’’ or chronic urate nephropathy is yet another condition associated with obesity. Histological analysis of the kidney specimens from 75% to 99% of gout patients reveals arteriosclerosis, arteriolosclerosis, glomerulosclerosis, tubular atrophy, and dilation of Henle’s loops [24]. Interestingly, the consumption of high-fructose corn syrup has been associated with increased uric acid production, which may contribute to kidney stone formation [23,112]. Moreover, hyperinsulinemia can reduce the urinary excretion of uric acid since insulin stimulates the urate-anion exchanger and/or the sodium-dependent anion co-transporter in the brush border membranes of proximal tubules [112].

## 12. Obesity-Related Glomerulopathy: An Overview

ORG is a secondary form of focal segmental glomerulosclerosis (FSGS) that typically affects obese patients with a BMI > 30 kg/m^2^ [185]. This condition may manifest at birth, often accompanied by proteinuria and, in some cases, a history of premature birth. It was first described by Cohen in 1975 [186]. Long-term longitudinal studies have shown that ORG begins with early glomerulomegaly, followed by FSGS-like lesions. Compared to idiopathic forms of FSGS, ORG has a lower incidence of nephrotic syndrome, often presenting a silent clinical course, and histologically showing lower pedicle fusion [187]. The pathogenesis of renal disease is likely related to hyperfiltration and glomerular hypertrophy, both of which correlate with increasing body mass [188]. In the kidney, the number of nephrons is predetermined at birth. As body mass increases, each nephron’s workload also increases progressively. This results in a gradual elevation of glomerular capillary pressures, exceeding the renal blood flow itself, causing glomerular hyperfiltration. Renal vasodilation, partly due to afferent arteriolar dilation, amplifies the hydrostatic pressure difference between the blood and the urinary space [162]. This lack of self-regulation exposes the glomeruli to higher systemic pressures commonly seen in obese individuals. 

Renal biopsy is required to confirm nephropathy and is often performed in patients with high proteinuria levels and impaired renal function. Retrospective data from patients diagnosed with obesity-related glomerulopathy via kidney biopsy revealed that at least 50% were initially diagnosed with idiopathic FSGS. Interestingly, the most affected populations tend to be older Caucasians. Clinically, patients with ORG have a lower incidence of nephrotic range proteinuria, hypoalbuminemia, and edema than patients with idiopathic FSGS [187,189]. Clinical progression is generally slow or steady, although some subjects may present with rapid deterioration of renal function and ESRD [150]. In the assessment of renal insufficiency, the albumin/creatinine ratio in urine (uACR) (pathological if higher than 30 mg/g) has been proposed as a screening test. This measurement is performed annually in patients with type II-DM, high BP, or obesity as a standalone disease. However, observational studies have shown that young patients undergoing bariatric surgery may experience impaired, reversible improvements in renal function. Notably, significant reductions in proteinuria may not always correlate with histological improvement.

The predominant pathological features of ORG include glomerulomegaly and secondary focal segmental glomerulosclerosis [190]. These glomerular lesions tend to precede overt renal failure [191]. ORG also shows fewer segmental sclerosis features and reduced fusion of the podocyte’s pedicellar processes compared with idiopathic glomerulosclerosis, despite evident glomerulomegaly [187]. Morphologically, ORG is also characterized by mesangial cell proliferation and matrix accumulation. 

Furthermore, there is a reported correlation between the number and density of podocytes and certain markers of insulin resistance [192].

## 13. Obesity as a Risk Factor in Children with Kidney Transplants

In adults with ESRD, obesity often poses a significant barrier to renal transplantation. Additionally, obese and underweight children face challenges in receiving a kidney transplant, particularly from a living donor. Moreover, they have an elevated risk of mortality, although this risk is attenuated when adjusted for transplantation in a time-dependent Cox model. These data are drawn from a recent retrospective analysis involving children aged 2–19 years who initiated renal replacement therapy between 1995 and 2011 in the U.S.A. [193].

Although many transplant centers around the world use a specified BMI value as a relative or absolute contraindication to kidney transplantation [194], this practice is controversial. Indeed, the effects of obesity on kidney transplant outcomes are still unclear [195]. While some researchers have not detected any significant adverse effects of obesity on kidney transplant outcomes [196], except for an increased occurrence of surgical wound complications or delayed graft function [197,198], conflicting studies have highlighted its negative impact on long-term renal graft and patient survival, often accompanied by an increased incidence and severity of post-operative complications [199]. A recent systematic review and meta-analysis [200] showed significantly higher odds of long- and short-term mortality and acute graft versus host disease in children with obesity; in contrast, no significant differences were shown between patients with and without obesity in terms of acute rejection. One more recent meta-analysis and systematic review [201] of three studies showed that pre-transplant sarcopenic obesity was associated with higher overall short-, intermediate-, and long-term mortality. Even a very recent cross-sectional study [196] of a limited number of patients (50 subjects aged 10 to 15 years) showed that obese children/adolescents had reduced graft survival. In addition, it is possible that individual characteristics, etiology of renal disease, type of transplant, transplant waiting time, differences in immunosuppressive therapy, and other factors may have justified this increased mortality in young obese transplant recipients [202,203,204]. Other conditions, such as complicated transplant surgery and ischemia time, might also play a role in inducing worse outcomes in patients with obesity [205]. Hence, these findings could not be directly translated to therapeutic interventions such as weight management prior to transplant, as there is currently a lack of comprehensive literature data on the clinical efficacy of such interventions and on the real advantages of weight modifications in terms of the consequences on post-transplant outcomes and on child growth in the long term. Overall, the existing literature does not appear to justify the use of high BMI as an absolute contraindication to pediatric kidney transplants due to mixed results on the impact of obesity on short- and long-term outcomes [206]. Future clinical trials are certainly expected to investigate the impact of pre-transplant management of body weight in obese children with ESRD on post-transplant outcomes and to establish guidelines on the management of obesity-associated post-transplant outcomes in children/adolescents [200]. Certainly, careful and accurate patient selection is important by recommending and verifying pre-transplant body weight reduction in order to reduce the rate of post-transplant complications and improve patient and transplant survival [196].

Children who received kidneys from obese living donors (BMI > 30), as opposed to non-obese living donors (BMI < 25), had lower glomerular filtration rates, a higher rate of allograft dysfunction, and distinct metabolic outcomes, such as elevated serum cholesterol levels and more severe hyperuricemia [207]. Furthermore, obesity increases the risk of short- and long-term adverse events in donors. While some researchers have reported no differences in kidney function between obese and normal-weight donors [208], others have documented an augmented rate of CKD after nephrectomy and an increased risk of developing proteinuria and CKD following unilateral nephrectomy [133,209]. Obese living donors have an increased risk of surgical wound complications [210,211] and of developing ESRD [212]. Hence, during the initial pre-transplant evaluation, all potential donors should be assessed for BMI and comprehensively evaluated for associated comorbidities, encompassing hypertension, hyperlipidemia, impaired glucose test tolerance, microalbuminuria, cardiovascular disease, sleep apnea, and liver disease [208].

## 14. Prevention and Management

**Figure 3:**
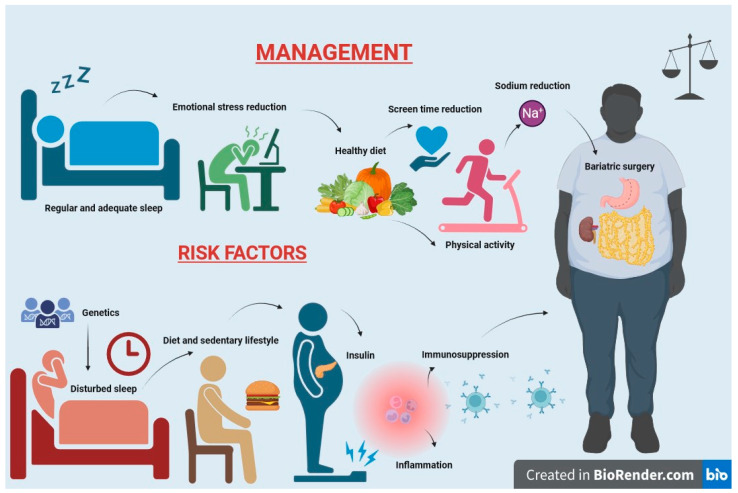
Risk factors and management of obesity in children with CKD.

Weight loss programs for overweight patients with kidney disease have documented a 30–50% reduction in proteinuria, although no significant improvement in renal function has been observed [213,214,215]. The prevention of pediatric obesity is of paramount importance and can be achieved through the promotion of a healthy diet, regular physical activity, and a healthy lifestyle (Figure 3). The principles governing the management of obesity are equally applicable to its prevention. 

The Pediatric Renal Nutrition Taskforce [216] for the management of obesity and metabolic syndrome in children with CKD stages 2–5 on dialysis and after kidney transplantation recommends a comprehensive multicomponent intervention that includes a nutrition care plan, physical activity prescription, and behavioral modification to reduce BMI and improve components of the metabolic syndrome. In this population, the use of anti-obesity medications is not recommended because there are no randomized controlled trials of these drugs in children or adults with kidney disorders. However, in obese adolescents with type 2 DM, anti-obesity pharmacotherapy could be considered [217]. Instead, weight loss surgery may be considered in a selected subgroup of children when all other interventions have failed [216].

While there is not a one-size-fits-all diet plan, some critical diet modifications, such as reducing excessive energy intake by limiting sugar and saturated fat intake (favoring unsaturated fats, particularly omega-3 fats), encouraging fiber, fruits, vegetables, and whole grain intake, should be considered. These dietary adjustments should be tailored to the individual’s age and CKD-appropriate requirements and appear to be suitable for preventing obesity in CKD children.

Physical activity should be adapted according to age, CKD stage, and comorbidities [218]. It is also advisable to minimize screen time and other positive psychosocial influences, such as family meals, mindfulness during eating, stress management, and emotional well-being, to prevent the onset of obesity [216]. Finally, a recent study by Liu et al. suggests that for some individuals, the problem may have its roots in childhood. Hence, prevention strategies may need to commence early in life, considering that some childhood factors, including adiposity, type 2 DM, low socio-economic status, and cardiorespiratory fitness (especially in females), may contribute to the CKD risk in adulthood [219].

Beyond that, certainly, RAAS inhibition has a renoprotective effect in individuals with ORG, and the use of some classes of hypoglycemic agents, such as sodium/glucose cotransporter 2 inhibitors or glucagon-like peptide-1 receptor agonists, could have positive effects given their emerging pleiotropic effects, although their use is not yet authorized in children [220,221].

## 15. Conclusions

In this review, we have examined the impact of childhood obesity on the kidney from an epidemiological, pathogenetic, clinical, and pathological perspective, with the aim of providing pediatricians and nephrologists with the most current data on this topic. 

The epidemiological trend of childhood obesity is reaching pandemic proportions, constituting one of the major public health problems. It is associated not only with a myriad of cardiovascular diseases but with the potential involvement of every organ, and the kidney is one of those most involved with repercussions in both the pediatric period and later adulthood. CKD is on the rise and is closely linked to the increasing incidence of obesity. Children and adolescents are also at risk for early kidney injury and the development of CKD. While the impact of obesity on children with chronic kidney failure is not as well documented as in adults, recent studies indicate a similar trend in both populations. Obesity contributes both directly and indirectly to the development of CKD and is also strongly related to the two most common causes of ESRD, namely DM and hypertension. 

Body adiposity has a heritable component, but pinpointing the genes responsible for common forms of obesity has been a challenging endeavor. Some genetic alterations in specific molecules, especially in children, can lead to obesity, such as those affecting leptin, pro-hormone convertase 1/3, congenital melanocortin-4 receptor deficiency, and GNAS gene mutations. The neurohormonal mechanisms underlying obesity have been extensively studied. Cytokines produced by the adipose tissue of obese patients induce adaptive or maladaptive responses in renal cells, impairing glomerular hyperfiltration. Now we can talk about an adipose–renal tissue axis with the involvement of complex neurohormonal, immunologic, and metabolic mechanisms. These could represent targets for the development of new therapies to improve the prognosis of childhood obesity and kidney disease.

Many adipokines, including leptin, adiponectin, vascular endothelial growth factor, angiopoietins, and resistin, play crucial roles in the accumulation of extracellular matrix, ultimately leading to renal fibrosis. Hypertension, even in the pediatric population, is a known risk factor for renal disease progression, and it is likely a major cause of renal dysfunction in obese patients, although it is not the sole hemodynamic cause. Masked hypertension is also a strong independent predictor of left ventricular hypertrophy, possibly due to hyperactivation of the renin–angiotensin–aldosterone system in these patients. Insulin resistance, either alone or in combination with hyperleptinemia, activates the SNS, causing vasoconstriction and reduced renal blood flow, leading to RAAS activation and water and sodium retention. Proteinuria, sleep apnea syndrome, and low serum levels of 1,25-(OH)_2_D_3_ also seem to play crucial roles. The study of new multiple markers will prove valuable in nephrology, offering different insights into lesion localization, involvement of inflammation, and system participation. Prominent among these markers, both well-established and recently discovered, are neutrophil gelatinase-associated lipocalin and the renal damage molecule KIM-1.

Lastly, chronic hyperfiltration potentiates renal damage, with increased protein loss, leading to glomerulomegaly, cellular remodeling, and fibrotic scarring. These potential mechanisms between obesity and kidney disease need to be further investigated, especially in children, in order to allow the initiation of preventive strategies aimed at halting the development of CKD. ORG is described as a secondary form of FSGS occurring in obese patients with a BMI > 30 kg/m^2^, often from birth, presenting with proteinuria, and possibly a history of premature birth. ORG is characterized by less segmental sclerosis and reduced fusion of podocyte pedicle processes compared with idiopathic glomerulosclerosis, despite overt glomerulomegaly. 

The effects of obesity on kidney transplant outcomes remain unclear. While some researchers have not identified significant adverse effects of obesity on kidney transplant outcomes, except for an increased risk of surgical wound complications, children receiving kidneys from obese living donors (BMI > 30) compared with those from non-obese living donors (BMI < 25) have shown a lower glomerular filtration rate, higher rates of allograft dysfunction, and different metabolic outcomes (elevated serum cholesterol and more severe hyperuricemia). Therefore, careful anthropometric evaluation of potential donors at the beginning of the pre-transplant assessment is warranted. 

Many aspects of disease progression in obese children remain to be elucidated. Nevertheless, recent scientific advancements in nephrology offer new perspectives, including genetic factors, comorbidities, and novel biomarkers. If investigated early, these factors could improve the prognosis of young patients through innovative diagnostic, preventive, and therapeutic strategies.

## Figures and Tables

**Table 2:** Potential mechanisms of kidney damage induced by obesity. Table adapted from “Obesity and chronic kidney disease: prevalence, mechanism, and management” [164].

Factor	Obesity Kidney-Related Damage
**Hemodynamic factors**	Glomerular hyperfiltration [165,166,167] Increased podocyte injury [104] Glomerulomegaly and glomerulosclerosis [104] Excessive tubular sodium reabsorption [168,169,170] Increased sympathetic activity [104] Increased renin–angiotensin–aldosterone system (RAAS) activity [170]
**Metabolic effects**	Abnormal lipid metabolism [64,77,171] Adipokine dysregulation [78,82,86,87,88,89,90,92,93,95,100,172,173] Increased insulin resistance [99,172] Increased inflammation [99,172] Increased oxidative stress [171,174,175]
**Lipid nephrotoxicity**	Excessive renal fat accumulation [174,175,176] Glomerular and tubular cell injuries [171,175,176] Mitochondrial dysfunction, oxidative stress and inflammation [171,175,177] Increased free fatty acid toxicity to proximal tubular cells [171,177]

## Data Availability

Not applicable.
